# Adoptive Cell Therapy Targeting Neoantigens: A Frontier for Cancer Research

**DOI:** 10.3389/fimmu.2020.00176

**Published:** 2020-03-05

**Authors:** Zhidong Wang, Yu J. Cao

**Affiliations:** State Key Laboratory of Chemical Oncogenomics, Key Laboratory of Chemical Genomics, Peking University Shenzhen Graduate School, Shenzhen, China

**Keywords:** adoptive cell therapy, CAR-T, TCR-T, neoantigen, neoantigen screening

## Abstract

Adoptive cell therapy (ACT) is a kind of immunotherapy in which T cells are genetically modified to express a chimeric antigen receptor (CAR) or T cell receptor (TCR), and ACT has made a great difference in treating multiple types of tumors. ACT is not perfect, and it can be followed by severe side effects, which hampers the application of ACT in clinical trials. One of the most promising methods to minimize side effects is to endow adoptive T cells with the ability to target neoantigens, which are specific to tumor cells. With the development of antigen screening technologies, more methods can be applied to discover neoantigens in cancer cells, such as whole-exome sequencing combined with mass spectrometry, neoantigen screening through an inventory-shared neoantigen peptide library, and neoantigen discovery via trogocytosis. In this review, we focus on the side effects of existing antigens and their solutions, illustrate the strategies of finding neoantigens in CAR-T and TCR-T therapies through methods reported by other researchers, and summarize the clinical behavior of these neoantigens.

## Introduction

Cancer is a kind of genetic disease that is caused by the accumulation of gene mutations. The genetic mutations within tumor cells lead to changes in the expressed proteins, and these changes control the process of transformation from healthy cells to tumor cells (1). In addition, these mutations provide, to some extent, numerous peptides that do not exist in normal cells, which makes them possible targets for the elaboration of an integrated neoantigen screening system, enhancing the development of adoptive cell therapies. By contrast, the immune system acts similar to an active soldier, who can respond to infections, search for and destroy diseased targets, such as pathogens or tumor cells (2). Neoantigens are derived from the genetic alteration of somatic cells, and can be targeted by the immune system to control malignancies (3). The last 30 years have witnessed the rapid development of adoptive cell therapy, as two critical parts of immunotherapy, CAR-T therapy, and TCR-T therapy, hold the promising possibility to cure cancer (4). Limited by severe adverse effects, the two kinds of immunotherapies need to be improved, and the screening and application of neoantigens is an effective way to enhance the specificity of CAR-T and TCR-T and minimize their side effects. We will now illustrate the occurrence, development and application of adoptive cell therapy and its side effects.

## Adoptive Cell Therapy

Adoptive cell therapy (ACT) is a kind of cancer treatment that endows T cells with the ability to recognize and kill cancer cells through gene engineering. To some extent, the manipulation of ACT strengthens or alters the intrinsic immune capacity and exploits its efficiency in the treatment of cancer disease. The application of TCR-T therapy in preclinical studies is due to the structure and function of the TCR. T cell receptors in T lymphocytes are important in the immune response, and different TCRs have different functions; for example, TCRs in cytotoxic T cells help to kill infected or abnormal cells, while TCRs in regulatory T cells help to inhibit responsiveness, and the specificity of these cells is governed by the TCRs (5). In 2006, Steven Rosenberg first reported the treatment of metastatic melanoma with TCR-T therapy and found that lymphocytes engineered to express TCRs that could recognize melanocyte-differentiating antigen (MART-1) had very positive effects in the treatment of the disease (6), which provides a new choice of cancer therapy. To our surprise, the advent of CAR-T occurred much earlier than that of TCR-T. In 1989, Eshhar et al. first combined scFv with the ζ chain of CD3 to make a recombinant and then transfected it into T cells; since then, CAR-T has come to our vision (7). These antibody-derived scFv-targeting chimeras could work in an antigen-dependent, HLA unrestricted way, which means that their use is not subject to the downregulation of restricting HLA molecules in tumor cells. With more and more effort put into cancer adoptive cell research, CAR-T therapy has gone through four generations: first generation CARs contain only a CD3ζ signaling domain, and second-generation and third-generation CARs hold one or more costimulatory domains (costim), respectively (8). To enhance the persistence and amplification of CAR-T cells, the TRUCT T cell, also called the fourth-generation CAR-T cell, has been constructed through the addition of a constitutive or inducible expression cassette expressing each kind of cytokine, which is released by the CAR-T cell to modulate the T cell response (9). However, TCR-T therapy is not clearly classified according to generations.

## Side Effects of Adoptive Cell Therapy and Their Solutions

### Side Effects of Adoptive Cell Therapy

Although effective responses have been observed in adoptive cell therapy, adverse effects have become a Gordian knot in many trials. Patients treated with lymphocytes modified with high-affinity TCR-T cells that target MART-1 or gp100 exhibited severe destruction in normal tissues where melanocytic cells were present, including the skin, eyes, and inner ears, which was due to the expression of MART-1 in normal cells (10). Patients with metastatic colorectal carcinoma treated with TCR-T cells targeting CEA exhibited severe inflammatory colitis, possibly because CEA is expressed in the normal mucosa of the colon (11). Three of nine patients treated with MAGE-A3-specific TCR-T cells experienced mental disturbances, and two of them died of leukoencephalopathy (12). The adverse effects of CAR-T therapy are similar to those of TCR-T therapy and include CAR-T cell-related encephalopathy syndrome (CRES), off-tumor effects and acute respiratory distress syndrome, which are due to on-target off-tumor recognition and killing; in addition, cytokine release syndrome (CRS) is the most frequent side effect of CAR-T therapy (13–15).

### The Solutions

**Figure 1:**
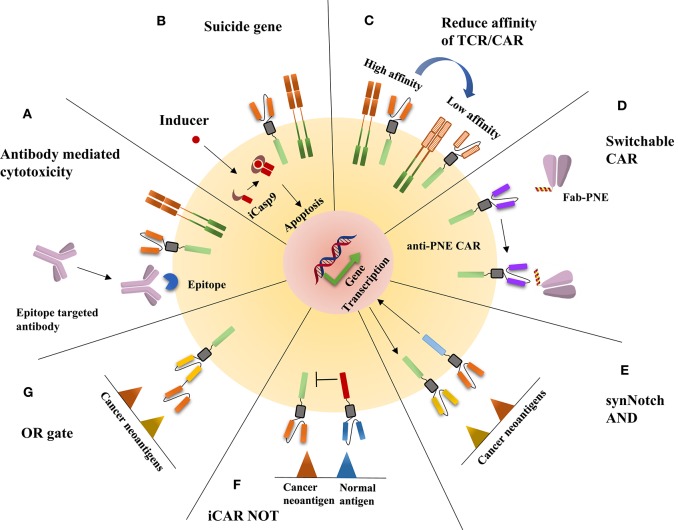
The illustration of the disposition of adoptive cell therapy side effects. **(A)** An epitope is expressed on CAR-T or TCR-T cells, which can be recognized by epitope-targeted antibodies, thus leading to CAR-T or TCR-T cells being killed through antibody-mediated cytotoxicity. **(B)** The addition of a dimerizing drug activates iCasp9 signaling and leads to apoptosis. **(C)** The reduced affinity of TCR/CAR can enhance specificity and reduce off-tumor on-target cytotoxicity. **(D)** The construction of switchable CAR is an effective method to reduce the side effects of CAR-T therapy; the strategy is to separate the antigen-binding domain from the signal transduction domain through a peptide neoepitope (PNE) that works as a bridge between the antigen-binding domain and the signal transduction domain. **(E)** On binding one tumor antigen, the synNotch receptor undergoes a conformational change that leads to the release of a transcription factor, which in turn drives the expression of a CAR-T antigen for another inhibitory antigen. **(F)** Inhibitory CAR (iCAR) dampens the T cell response when a normal antigen is encountered. **(G)** OR gate CAR is comparable to bispecific CARs.

Different cell therapies have different solutions to eliminate side effects (Figure 1). For TCR-T therapy, a switch is inserted into TCR-T cells, such as the inducible caspase 9 safety switch, herpes simplex virus thymidine kinase, or a truncated human epidermal growth factor receptor, which makes the TCR-T cells lethally sensitive to the related ligands; when side effects occur, the reaction can be terminated through the administration of the related ligand (16–18). In addition, reducing the affinity is also an effective method, because, as with many other cell surface receptors, αβ TCRs bind to peptide-MHC (pMHC) complexes with a very low affinity (~1–50 μM) (19); thus, if TCRs have higher affinity with their cognate, they might enhance the ability to recognize the relatively low-affinity antigen in normal cells, which will cause severe side effects. To address the side effects of CAR-T therapy, numerous approaches have been developed to manipulate the activity and depletion of adoptive T cells. These approaches include all methods applied in TCR-T cell side effect control; for example, to rapidly ablate CAR-T cells, inducible caspase (iCasp9) is expressed on CAR-T cells, and, similar to the treatment of TCR-T cell side effects, this is done with the addition of a dimerizing drug that activates iCasp9 signaling and leads to apoptosis (20, 21). Other suicide genes, such as epitope tags, are also used to control CAR-T cell reactions (22). In addition, the affinity altering method is applied in CAR-T cell side effect control (23). Furthermore, switchable CARs have been designed to increase the safety and manipulate the activity at human will, reducing the cytotoxicity without the deletion of programed cells. The strategy is to separate the antigen-binding domain from the signal transduction domain through a peptide neoepitope (PNE). The PNE is designed to contact an antibody that recognizes and binds a specific antigen on cancer cells, while the PNE can be recognized by antibodies on the adoptive T cells and thus acts as a bridge between the antigen-binding domain and the signal transduction domain (24, 25). In addition, logic gate CAR, using AND, NOT, and OR logic gates, can also increase specificity and reduce side effects. The most prevalent AND gate CAR is synNotch CAR (26), which imitates the Notch signaling pathway activation mechanism; this strategy contains a synthetic Notch receptor (synNotch) that releases a transcription factor once the receptor recognizes its cognate, in turn driving the expression of a CAR specific for a specific antigen. The NOT gate CARs can distinguish normal cell antigen from cancer cell antigen through the coexpression of an inhibitory CAR (iCAR) that dampens the T cell response when normal cell antigen is present (27). The OR gate CAR is similar to the bispecific CAR, which integrates CD3ζ and the costimulatory domain with two CARs recognizing different antigens (28).

These safety mechanisms may have limitations, both in TCR-T and CAR-T immunotherapies, such as a relatively slow induction efficiency, and inherent leakiness can lead to residual TCR/CAR expression in the absence of the inducer (29), which limits the wide application of adoptive cell therapy and threatens the safety of patients. The most crucial event in the development of ACT is to find high-specificity neoantigens, which will reduce the tedious safety control methods and help to make ACT a widely applied therapy. The potential advantages and disadvantages to apply TCR-T or CAR-T based on neoantigens for cancer therapy were discussed in Table 1. Although there are tremendous obstacles and undiscovered mechanisms in the immune system, efforts in related studies make a difference, and some methods are available for the discovery of neoantigens.

**Table 1:** Advantages and disadvantages to use TCR-T or CAR-T based on neoantigens.

**Adoptive cell therapy**	**Advantage**	**Disadvantage**
TCR-T	1. Designed to detect intracellular antigens with a high mutation rate 2. Low affinity but high antigen sensitivity 3. Natural protein with low immunogenicity	1. MHC dependent antigen detection, with limited patient applicability 2. Mispairing with endogenous TCRs could cause non-specific efficacy 3. Dynamic variation of neoantigen landscape in different patients 4. Difficult to identify neoantigens in low mutation rate cancers
CAR-T	1. MHC independent antigen detection of soluble or cell surface antigens 2. High antigen affinity 3. Modular design enables precise control neoantigen response 4. Recognize not only proteins but also carbohydrates and glycolipids that arise during tumorigenesis	1. Limited neoantigen recognition 2. On-target CAR-T cell activation in the presence of soluble antigens 3. Ability to recognize cell-surface antigen may be blocked by the presence of competing soluble antigen 4. Unnatural protein may be immunogenic 5. The heterogeneity of tumor cells

## Methods to Screen Neoantigens

### Whole-Exome Sequencing (WES) Combined With Mass Spectrometry (MS)

#### Workflow of WES/MS

**Figure 2:**
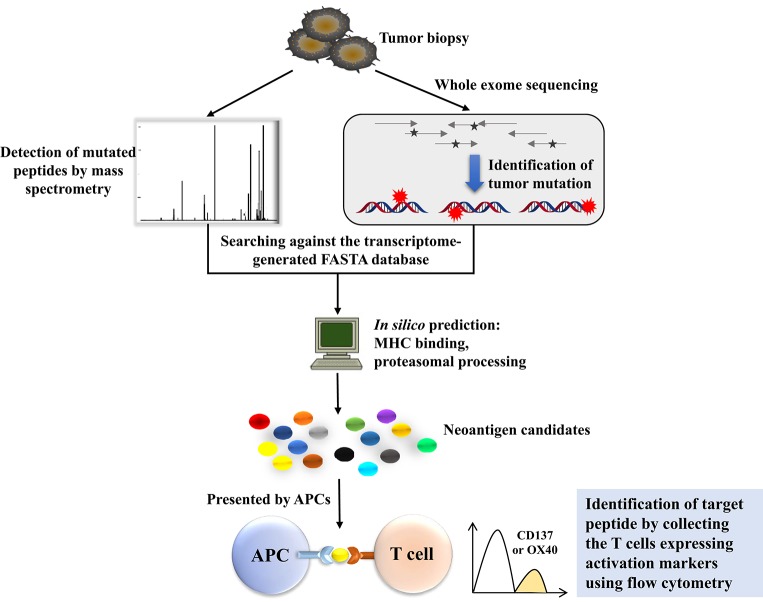
The workflow of neoantigen screening using whole-exome sequencing (WES) combined with mass spectrometry (MS). WES is conducted to identify the tumor-specific mutations, together with mass spectrometry-based mutated peptide detection, to compare the mutated proteins with those in the transcriptome-generated FASTA database. Mutated proteins will be predicted *in silico* to narrow down the target mutations. Predicted peptides can be expressed by a patient's APCs, where they are processed and presented in the context of a patient's MHC. The coculture of the patient's autologous T cells with these APCs can be used to identify the mutations processed and presented by APCs. The identification of individual mutations for tumor recognition is applicable because T cells express activation markers such as OX40 or CD137 when they recognize the cognate target antigen.

Human tumor cells typically harbor remarkable numbers of somatic mutations, and cancer genomics can be mined with sequence technology to gain insight into the landscape of tumor-specific mutations from which such neoantigens may derive (30). Recent studies have indicated that if these mutations are translated to proteins and presented by major histocompatibility complexes, peptides containing these mutations should be recognized as neoantigens by the adaptive immune system as they are non-self-proteins (31). We need to determine which mutated genes are expressed and whether these proteins are present on the surface of tumor cells by the MHC molecule. The combination of WES and MS exactly solves these problems (Figure 2). As is well-known, classic cDNA sequence technology is labor- and time-intensive, and there are some obstacles in identifying high-GC sequences and low-copy transcripts (32). However, recent technological advancements in next-generation sequencing (NGS) and tandem MS make it possible to acquire detailed sequences for mutations in some kinds of cancers, which provides a strong foundation for screening and exploring neoantigens in cancer research (3). The workflow is that, first, WES should be performed to identify the tumor-specific mutations and select for high-confidence mutations through RNA-seq-based variant frequency that overlap with the exome-based variants. Next, MS analysis should be conducted, and the transcriptome-generated FASTA database should be searched to reduce the workload and raise efficiency. When conducting MS, what needs to be done first is to make an enrichment with an HLA-1 affinity column; thus, the HLA-1 correlated proteins are isolated and identified. Next, mutated proteins will be predicted using the NetMHC-4 or NetMHCpan algorithm (33) to narrow down the target mutation. The combination of WES and MS has become a powerful weapon for exploring neoantigens in tumor immune therapy. Andrea Garcia-Garijo further applied this method to make a difference in mining neoantigens in melanoma and renal cell carcinoma, and gave detailed illustrations of the identification of tumor-specific non-synonymous mutations and the evaluation of immunogenicity of candidate neoantigens (34).

#### The Applications of WES/MS

Yadav et al. combined MS and WES to predict immunogenic tumor mutations. They applied this method in two widely used murine tumor models. They identified a total of 1,300 amino acid changes in these two models, 13% of which were predicted to bind MHC class I molecules. Some of these proteins were confirmed by mass spectrometry, and the vaccination of mice with these mutated proteins confirmed that the mutated proteins were immunogenic, as predicted by the immunogenic peptide yielding therapeutically active T cell responses (35). However, tumor models cannot simulate the complex environment of actual tumors, therefore the Matthias Mann group developed a high-sensitivity method to conduct an analysis of 25 human native tumor specimens. They used high-sensitivity MS to analyze exome statistics, and through this method, they discovered tumor-specific neoantigens in selected patients that were validated by the evidence of potent patient-derived neoantigen-specific antitumor immune responses (36). Gros et al. used WES to find non-synonymous mutations in metastatic gastrointestinal cancer with low mutational burden, and further synthesized these mutated peptides or tandem minigenes to pulse into Antigen Presenting Cells (APC), which could enrich the circulating CD8^+^/PD-1^+/hi^ and CD4^+^ PD-1^+/hi^ T cells harboring neoantigen reactive TCR from cancer patients (37).

#### Obstacles to This Strategy

There are some unavoidable obstacles that hinder the application of these methods to screen neoantigens in all kinds of cancer cells. Different cancers contain different numbers of mutations, and cancers with a high mutation rate often show good responses to immunotherapy; for example, melanoma and lung cancers are more susceptible to immune therapies, including checkpoint block and adoptive cell therapy (32). However, it is difficult to detect neoantigens in cancer cells with a low mutation load, although neoantigens also occur (38, 39), but in this condition, this method cannot distinguish them from normal peptides. In addition, most neoantigens are unique to one special patient, although there are some neoantigens, such as the MYD88^L265P^ mutation, the histone 3 variant H3.3^K27M^ mutation, and the KRAS^G12D^ hotspot-driver mutation, that are present in several patients (40–42). Even in the same cancer entity, the distribution of neoantigens is heterogeneous, which hinders the application of single anti-cancer drugs and creates an intractable problem in cancer treatment. The accuracy of the MHC/HLA binding prediction algorithm also limits the screening approach, which has not been thoroughly examined for MHC class II and infrequent HLA alleles (32). In addition, many factors influence the expression of T cell epitopes on the cell surface; for example, the multiple forms of the proteasome determine the number of peptides that truly proceed and are presented by MHC (43, 44). These outcomes of sequencing and MHC binding predictions are not convincing enough to make these candidate peptide neoantigens; only if the targeting T cells are activated by these antigens can we verify those mutated peptides as neoantigens. Consequently, some researchers have explored a method to identify the antigenicity of these peptides; they pulsed the peptides into APC cells, such as autologous dendritic cells or B cells, cocultured these cells with autologous T cells, and then detected IFN-γ secretion or other activation markers, such as OX40, CD25, and CD137, to judge whether the antigen induces an adaptive immune response (45). Parkhurst et al. demonstrated that this method worked. They designed one minigene encoding 25 amino acids, in which the mutated amino acid located in the middle, and a tandem minigene containing 12–24 different minigenes was cloned into an expression vector to evaluate these candidate peptides simultaneously. In that way, 25 amino acid candidate peptides were synthesized, in the middle of which the mutation was located, pulsed together into APCs and coincubated with autologous T cells (46).

### Neoantigens Screening Through an Inventory-Shared Neoantigen Library

**Figure 3:**
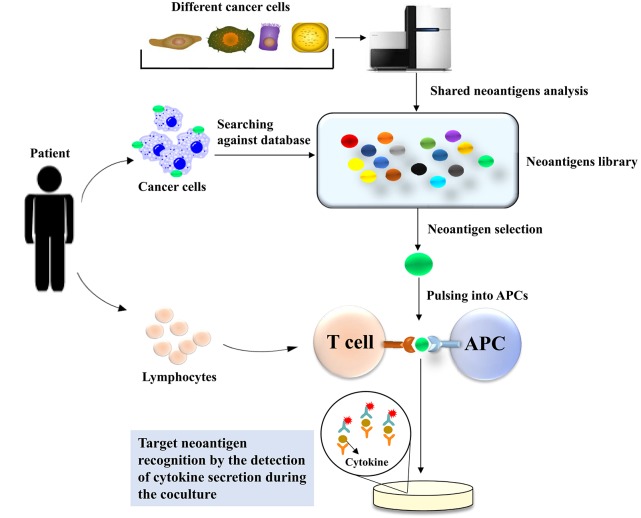
Neoantigen screening through an inventory-shared neoantigen library. The TCGA and COSMIC databases are used to mine high-frequency mutant genes in nine types of human malignant solid tumors to construct the neoantigen library. Data from the patient who underwent targeted sequencing were compared with those in the neoantigen library to identify the specific neoantigen in the patient's tumor cells. The selected neoantigen will be pulsed and presented by APCs in the context of the patient's MHC. The coculture of the patient's T cells with the APCs can be used to identify the neoantigen presented by the APCs. The detection of immunogenic neoantigens for tumor recognition is performed by ELISPOT-based cytokine detection secreted from activated T cells when they recognize the target neoantigen.

Recently, a Chinese research group devised another kind of method to screen neoantigens in patients who suffer from cancers (47), with the expectation to identify neoantigens in a timely and convenient manner. They created an off-shelf neoepitope peptide library. They first mined high-frequency mutant genes in nine types of human malignant solid tumors with the TCGA and COSMIC databases and found genes with frequencies >10% in the COSMIC database. There were 21 mutant genes with frequencies >10%; among these mutant genes, 29 hotspot mutations were selected as candidate targets to build the shared neoantigen peptide library, which covered 9.49~89.56% of cancer patients in the TCGA database, with a median coverage of 23.04%. They selected the high-frequency HLA-A class I gene product subtypes, HLA-A*11 (A*1101), HLA-A*02 (A*0201, A*0203, and A*0206), and HLA-A*24 (A*2402), and determined the possibility of these candidate peptides binding to HLA-A with five different algorithms: BIMAS, IEDB, NetMHC3.4/NetMHC4.0, NetCTL1.2, and SYFPEITHI. After the analysis of these algorithms, 44 shared neoepitope peptides were selected for peptide synthesis. In the clinic, the patients who underwent targeted sequencing were retrieved against the neoantigen library, and the researchers found that the mutations in 13 patients corresponded with the shared neoantigen peptide library. In addition, HLA-A alleles were also matched, and then immunogenic neoantigen identification was conducted by detecting the secretion of IFN-γ using Cytometric Bead Array and ELISPOT. Eventually, immunogenic neoantigens were identified in six patients through the neoantigen peptide library (Figure 3).

This method is more convenient and time-saving than WES combined with MS, but there are also some problems that need to be pointed out. First, the capacity of the shared neoantigen database must cover as many candidate peptides as possible to incre ase the accuracy of the identification of neoantigens. Second, the accuracy of the prediction algorithm also limits the outcomes, as with the previous method, WES combined with MS. However, with an increasing number of neoantigens being identified, this kind of method is a promising approach to neoantigen screening.

### Neoantigen Screen via Trogocytosis

**Figure 4:**
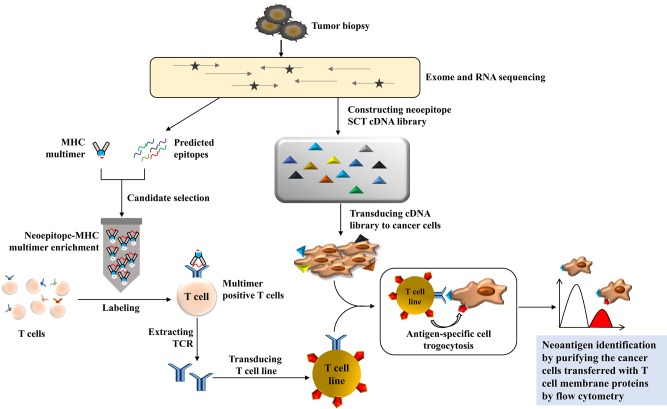
The illustration of neoantigen screening via trogocytosis. The private mutations are identified by exome and RNA sequencing from tumor samples, and the neoepitope ligand can be predicted and presented by an MHC multimer panel to be applied to the patient's autologous T cells. The gene of neoepitope-reactive TCR can be verified and transduced into a T cell line as an effector cell line. Meanwhile, the neoepitope single-chain trimer (SCT) cDNA library can be generated and transduced into K562 or other cancer cell lines to construct the target cell library. The coculture of effector cells with the target cell library will be used to identify the neoepitope because the membrane protein from the T cell line will transfer to the specific target cell whenever the neoantigen matches the T cell with a specific TCR. After two rounds of flow cytometry-based cell sorting, the neoantigen for tumor recognition can be isolated based on the specific membrane protein transferred to neoepitope-transduced target cells.

Li et al. developed an entirely fantastic method to discover T cell antigens through trogocytosis (48). Trogocytosis is a biological phenomenon that happens during cell conjugation by which cells share membranes and membrane-associated proteins (49). It has been reported that trogocytosis is a bidirectional physiological activity, but Li et al. found that target cells with supraphysiological levels of epitopes can extract membranes and membrane-associated proteins from interacting T cells; this phenomenon can be tracked by the acquisition of T cell membrane proteins. Using this characteristic of T cell-target cell reactions, they developed a TCR ligand discovery platform that could distinguish target cells that present genetically encoded epitopes cognate to orphan TCR-transduced T cells with fluorescence-activated cell sorting from a target cell library. They established a Jurkat cell line expressing F5-TCR or 1G4-TCR and K562 cells expressing their cognate single-chain trimer of HLA-A2/MART1 or A2/NYESO1. They coincubated these two kinds of cell lines and found that trogocytosis occurred from T cells to target cells and was scaled with pMHC (peptide-MHC) density; therefore, they labeled the orphan TCR of T cells and coincubated with cognate target cells, and they sorted the target via FACS with high specificity. Next, they determined whether this platform can be applied in a real tumor model; that is, whether or not this method can identify the cognate neoepitope for a tumor-infiltrating lymphocyte-derived orphan TCR from a custom library of privately mutated, subject-specific neoepitopes. They chose metastatic melanoma cells as the target cell. First, they identified private mutations through exome and RNA sequencing and then predicted which of these mutations generated neoepitopes that would be presented by HLA-A*02:01. A neoepitope-reactive TCR was isolated through a pMHC multimer panel. A neoepitope SCT cDNA library comprising 3,251 unique neoepitopes ranging from 8 to 12 amino acids in length was constructed, and K562 cells were transduced with this library. They then coincubated K562 cells with neo-TCR-transduced Jurkat cells and performed two rounds of sorting and neoepitope identification through next-generation sequencing. Eventually, they found the highest rank peptide was derived from ubiquitin-specific peptidase 7, and they verified the neoepitope by measuring the ability of the neo-TCR to induce cytotoxicity in the recognition of mutUSP7-K562 target cells (Figure 4).

This method is a new breakthrough in T cell antigen discovery. In comparison with pMHC yeast display (50), the method has some advantages. First, it does not need to produce orphan TCR protein reagents, and the orphan TCRs are expressed in their natural context. Second, the extent of trogocytosis can be controlled through the coexpression of CD8 molecules on the donor cells because it can increase the avidity between T cells and cognate target cells, which could be used to adjust the screening specificity. However, the neoepitope discovery is based on the exome sequence and RNA sequence, and prediction with bioinformatics tools, which has the drawbacks mentioned before, in addition to the construction of neoepitope libraries, is laborious and time-consuming, which limits the wide application of this method.

These different screening methods have their own characteristics, which are applicable to specific situations. The advantages and disadvantages of different approaches for neoantigen identification are listed in Table 2. In early research, serological analysis of recombinant expressed cDNA clones (SEREX) was demonstrated to be a useful method to detect tumor- and tumor-associated antigens in a variety of malignancies (51, 52). This kind of method utilizes antigen and antibody reactions to identify tumor antigens. Similar to ELISA, a tumor cDNA library first needs to be constructed and transfected into *Escherichia coli*; the expressing antigens are transferred to the membrane and then coincubated with patient serum antibody; and enzyme-conjugated anti-human IgG antibodies are used to generate a color reaction to identify the tumor-associated antigens. Compared to SEREX, these methods mentioned before not only can screen neoantigens but also, in a high throughput way, can improve the accuracy. Recently, Kula et al. developed another method for T cell epitope discovery, and the mechanism of this method is almost the same as that of Li, but they used lentiviral delivery of antigen libraries into target cells rather than an SCT library, and they coincubated these transduced target cells with T cells if the target cells triggered T cells to secrete granzyme B; the antigens transduced would be identified by next-generation sequencing (53). Joglekar et al. described the use of chimeric receptor called signaling and antigen-presenting bifunctional receptors (SABRs) in a cell-based platform for TCR antigen discovery. By connecting a MHC/peptide complex with CD3ζ-CD28 intracellular signaling domain, they devised a novel reporter system for antigen discovery to screen thousands of antigenic epitopes, and identified the targets recognized by public TCRs of known specificities (54).

**Table 2:** Advantages and disadvantages of different approaches for neoantigen identification.

**Approach**	**Advantage**	**Disadvantage**
Whole exome sequencing combined with mass spectrometry	High throughput Identify post-translational modification	Many false positives
Neoantigens screening through inventory-shared neoantigens library	Labor and time saving	Depend on the capacity of shared antigen database
Neoantigen screen via trogocytosis	Identify the neoantigen and TCR simultaneously	Hard to manipulate, especially construct SCT cDNA database and transduce into cancer cells

## Reported Neoantigens

The role of neoantigens in successful clinical activities is broadly accepted, although some important questions remain, including the size and quality of neoantigens across tumors, which dictates the capacity of neoantigens to induce T cell activation, and how neoantigens are sustained when T cell stress occurs as the landscapes of neoantigens in tumors are dynamic during tumor-infiltrating lymphocytes (TIL) interaction (55). Recent years have witnessed many promising neoantigens, whether by chance or with neoantigen screening technologies, and these neoantigens provide some insight into these questions (Table 3).

**Table 3:** Neoantigens for adoptive T cell therapy in clinical trials.

**Neoantigen**	**Disease**	**Intervention**	**Patient number**	**Phase**	**Country**	**NCT number**
EGFRvIII	Esophagus Cancer, Hepatoma Glioma, Gastric Cancer	CAR-T/TCR-T cells immunotherapy	50	I/II	China	NCT03941626
	Glioblastoma	CAR-EGFRvIII T cells	7	I	US	NCT03726515
	Recurrent Glioblastoma	EGFRvIII-CARs	24	I	US	NCT03283631
	Glioblastoma Multiforme	Anti-EGFRvIII CAR T cells	20	I	China	NCT02844062
	Residual or Recurrent EGFRvIII+ Glioma	CAR-EGFRvIII T cells	11	I	US	NCT02209376
KRAS mutant	Gastrointestinal Cancer Pancreatic Cancer Gastric Cancer	Anti-KRAS^G12D^ mTCR PBL	70	I/II	US	NCT03745326
	Pancreatic Cancer Gastric Cancer Gastrointestinal Cancer Colon Cancer Rectal Cancer	Anti-KRAS^G12V^ mTCR PBL	110	I/II	US	NCT03190941
Tn-MUC1	Advanced Esophageal Cancer	Anti-Tn-MUC1 CAR-T cells PD-1 knockout Engineered T cells	20	I/II	China	NCT03706326
	Intrahepatic Cholangiocarcinoma	Tn-MUC-1 CAR-T cell immunotherapy	9	I/II	China	NCT03633773
	Lung Neoplasm Malignant Non-small Cell Lung Cancer	Anti-Tn-MUC1 CAR-T Cells and PD-1 Knockout Engineered T Cells	60	I/II	China	NCT03525782
	Advanced Solid Tumor	Anti-CTLA-4/PD-1 expressing Tn-MUC1-CAR-T	40	I/II	China	NCT03179007
	Pancreatic Neoplasms	Dendritic cells pulsed with Tn-MUC-1/WT-1 peptides	30	I/II	Belarus	NCT03114631
	Hepatocellular Carcinoma Non-small Cell Lung Cancer Pancreatic Carcinoma	Anti-Tn-MUC1 CAR-pNK cells	10	I/II	China	NCT02839954
IDH1 mutant	Glioma	Dendritic cells	30	NA	China	NCT02771301

*EGFRvIII, epidermal growth factor receptor variant III; NA, not available; mTCR PBL, mutated TCR peripheral blood lymphocyte; Tn-MUC-1, Tn (GalNAcα1-O-Ser/Thr) glycoform of MUC1; WT, wild type; pNK cell, peripheral natural killer cell; IDH-1, Isocitrate dehydrogenase 1*.

### EGFRvIII

Epidermal growth factor receptor variant III (EGFRvIII) is the variant of EGFR in human tumors that is widely distributed (56). This variant results from a deletion and a mutation in the exon of EGFR, which create a tumor-specific and immunogenic neoantigen (57). Previous studies have demonstrated that in both human and mouse, EGFRvIII can induce the autoimmune response, and the EGFRvIII-specific antibodies could even be isolated from breast cancer patients (58, 59). Marcela Maus conducted a first-in-human study of the treatment of glioblastoma with anti-EGFRvIII CAR-T therapy (60); it is impressive that after the infusion of anti-EGFRvIII CAR T cells, the cancer was suppressed, without evidence of off-tumor toxicity or cytokine release syndrome. Shuangyin Han constructed chimeric EGFRvIII scFv-ICOS-CD3ζ (EGFRvIII CAR) using lentivirus and found that there was a robust increase in IFN-γ secretion after coincubating anti-EGFRvIII CAR T cells with EGFRvIII-expressing U87 cells; moreover, EGFRvIII CAR T cells inhibited the *in vivo* growth of EGFRvIII-expressing glioma cells in a xenograft mouse model regardless of intravenous or intratumor injection (61). Yu et al. transduced human NK cell lines NK-92 and NKL and primary NK cells with a lentivirus containing anti-EGFR CAR to evaluate the anti-GB efficacy; they found that intracranial administration of anti-EGFR CAR NK-92 cells resulted in efficient suppression of tumor growth and significantly prolonged the survival of tumor-bearing mice (62).

### KRAS Mutant

KRAS is a pivotal oncogene in numerous human cancers; is the upstream activator in many signaling pathways, especially the MAP kinase pathway; and determines the division and metabolism of cells (63). The predominant variants of KRAS are site mutations at codon 12, in particular G12D and G12V, which account for 60–70% of pancreatic cancers and 20–30% of colorectal cancers (64). Interestingly, there is a hypothesis that EGFR and KRAS mutations have functionally equivalent roles in lung tumorigenesis because mutations of both EGFR and KRAS are rarely found in the same tumors (65). Qiong J Wang isolated one T cell receptor with high affinity for the mutated KRAS variants G12V and G12D, transduced peripheral blood lymphocytes (PBLs) with these TCRs and found that these genetically engineered PBLs could recognize multiple HLA-A*11:01^+^ tumor lines that destroy these target cells; in addition, the adoptive transfer of these transduced PBLs could significantly reduce the growth of tumors in a xenograft model (66). Tran et al. identified a polyclonal CD8^+^ T cell from a patient with metastatic colorectal cancer which responded to KRAS G12D mutation tumor cells; objective regression was observed after adoptive transfer TILs specifically targeted KRAS G12D (67). As a driver mutation, the KRAS mutant is conceptually attractive since it is tumor-specific and biologically important to tumor progression and is likely to be expressed in all kinds of tumors (68), which makes the KRAS mutant a hot spot for adoptive cell therapy.

### MYD88 Mutant

MYD88 is a Toll-like receptor (TLR) adaptor protein, and ~90% of certain non-Hodgkin lymphomas (NHLs) have the Leu265Pro (L265P) mutation, which is a driver mutation (69). Signaling studies have shown that the mutation of MYD88 at site 265 triggers tumor growth by activating the NF-κB signaling pathway. As MYD88^L265P^ is a widely occurring and tumor-specific mutation in NHL, Nelde et al. predicted potential MYD88^L265P^-containing HLA ligands for several HLA class I restrictions *in silico*. Three HLA-B*07-restricted peptides and one HLA-B*15-restricted peptide were identified, and they found that MYD88^L265P^-derived peptides can induce mutation-specific and functional immune responses *in vitro* (41).

### IDH1 Mutant

Isocitrate dehydrogenase type 1 (IDH1) usually mutates in the development of a subgroup of gliomas (70). The arginine residue (Arg132) in the catalytic pocket tends to mutate during tumorigenesis, resulting in different enzymatic functions that catalyze the production of the oncometabolite 2-hydroxyglutarate (2-HG). In 2-HG metabolism-deficient patients, the excess accumulation of 2-HG will foster brain tumor growth. Dang et al. found that human malignant gliomas harboring IDH1 mutations resulted in an increase in 2-HG, which contributes to the formation and malignant progression of gliomas (71). There is a statistic showing that more than 70% of diffuse grade II and grade III gliomas carry the most frequent mutation, IDH1^R132H^ (72, 73). Theresa Schumacher found that mice whose MHC molecules were deficient and that were vaccinated with the human MHC class I/II with IDH1^R132H^ p123-142 experienced tumor regression (74). Michael Platten has shown that IDH1^R132H^ is an immunogenic tumor antigen that induces mutation-specific CD4^+^ T cell and antibody responses that are capable of controlling IDH1^R132H^-expressing tumor growth *in vivo* in MHC-humanized A2.DR1 mice after vaccination; because of its CD4^+^ T cell-dependent manner, IDH1^R132H^ is suitable for adoptive cell therapy (75).

### p53 Mutant

The identification and characterization of mutant p53 pR175H was conducted by Steven Rosenberg's group (76). Mutant p53 pR175H is a kind of neoantigen found in a subset of patients with cancer. p53 has been described as “the guardian of the genome” because of its role in conserving stability by preventing genome mutation; thus, the mutation of p53 has a serious adverse effect on normal cells and accelerates the process of carcinogenesis as a driver mutation. Lo et al. screened tumor-infiltrating lymphocytes for the recognition of mutated neoantigens in a patient with metastatic colorectal cancer in a HLA-A-dependent manner; they found that the minimal peptide epitope of pR175H was HMTEVVRHC, and the screened TIL also mediated the recognition of p53 pR175H^+^ colon, breast, and leukemia cell lines after transduction with a retrovirus encoding HLA-A*0201 (76).

### Tn-MUC1

The MUC1 membrane mucin is a high-molecular-mass glycoprotein encoded by the *muc1* gene, also called CD227, which was first identified through a monoclonal antibody binding experiment (77). It is well-known that MUC1 promotes cell growth and survival (78). Evidence shows that the overexpression of MUC1 is related to cell adhesion inhibition and the increased metastatic potential of tumor cells, especially in breast cancer (79). Although MUC1 in cancer cells is not directly produced by gene mutation, but its aberrant glycosylation and conformation changes are partly due to the mutation of other genes such as loss-of-function mutations in Cosmc gene in cancer cells (80). By confocal microscopy of immunostaining assay, Avery Posey found the Tn (GalNAcα1-O-Ser/Thr) glycoform of MUC1 (Tn-MUC1) expressed intracellularly in normal tissue such as human kidney, but at the cell membrane in several cancers. Based on that, his team developed an anti-Tn-MUC1 CAR-T cell that recognized the cancer-specific cell surface expressing Tn-MUC1, and demonstrated the target-specific cytotoxicity and suppression of tumor growth in xenograft models of T cell leukemia and pancreatic cancer (81). This finding demonstrates that Tn-MUC1 might be a potential target for cancer therapy. In early research, Wilkie et al. developed dual-targeted CAR-T cells by coexpressing ErbB2- and Tn-MUC1-specific CARs with respective costimulatory domain. They found that “dual-targeted” T cells kill ErbB2^+^ tumor cells efficiently, but their proliferation requires coincubation with the target cells with both Tn-MUC1 and ErbB2 expression (82).

In addition to the neoantigens mentioned above, there are many other antigens that have been discovered either by WES or by chance, such as the GAS7 mutant, CSNK1A1, and HAUS3 (83); these antigens deepen our understanding of tumorigenesis and development and can be developed as potential targets for cancer treatment. From a clinical perspective, preferred neoantigens would be formed by epitopes encoded by mutations that are shared across patients and, to reduce the risk of immune escape, locate to driver genes that are essential for tumor survival. On the other hand, identification of neoantigen-specific lymphocytes is also a promising and helpful way to enrich the treatment of cancer, Steven Rosenberg has developed an intact method to screen neoantigen-specific lymphocytes, they aim at tumor infiltration lymphocytes and use PD-1 as a biomarker to detect T cells that target neoantigens (84, 85). When the technology of neoantigen mining is well-established, the finding and application of neoantigens will save time and labor.

## Concluding Remarks

There is an increasing excitement in the field that ACT can be a potent new addition to the toolbox for cancer therapy. However, many of TCR-T/CAR-T cell trials were checked by safety concerns, highlighted by the occurrence of on-target and off-target adverse effects, although uncommon, has been fatal on occasions. While timely pharmacological intervention is effective in the management of a majority of adverse events but ACT can persist long term, along with any unwanted effects. on the other hand, the tumor-restricted expression of neoantigens driven by somatic mutation ensures the therapeutic generation of cell therapy reactivity against these antigens, which will not be associated with the toxicity in normal tissues, and considered to be the ideal and safe solution in ACT.

Neoantigens are derived from the alteration of genetics or virus infection, and they can be targeted specifically by the immune system to control malignancies. The evidence supporting the relevance of neoantigens in clinically successful immunotherapies is compelling and provides a strong rationale for the therapeutic targeting of these antigens. However, mounting evidence suggests that only a small fraction of neoantigens identified successfully with inherent difficulties such as tumor heterogeneity, accuracy and specificity of next-generation sequencing, as well as dynamic immune-editing landscape of neoantigens in tumors. With the development of technologies in whole-exome sequencing, inventory-shared neoantigen library and trogocytosis screening platforms, an increasing number of candidate neoantigens will be identified and determined to enhance the shared neoantigen database capacity, which will conceivably help to disclose the hidden secrets of tumors and neoantigens. Despite the rapid advances, enormous challenges remain for the future development of neoantigen-based adoptive cell therapy for wide clinical applications. So far, most clinical and preclinical studies have been focused on the T cell epitope mapping and screening, the feasibility of applying the strategies to B cell epitope with spatial configurations is to be demonstrated. It also remains challenging to identify and select the multiple immunogenic neoantigens from an individual tumor for enhanced therapeutic efficacy.

Rationally designed strategies to identify candidate neoantigens and to evaluate their immunogenicity are of vital interest to boost the safety and efficacy of ACT. Together with the advances in broad tumor immunogenomics sequencing technology as well as accurate and comprehensive *in silico* peptide prediction strategy, it will enable the identification of a target set of neoantigens for cancer immunotherapy. Additionally, neoantigens are not restricted to the application of ACT, and they can also make a difference in cancer vaccine targets and the understanding of cancer mechanisms. To avoid tumor escape, it will be necessary to combine the neoantigen-targeted ACT with checkpoint-blocking antibodies or autologous tumor cell vaccines in cancer patients to achieve a much higher response rate. With the increasing number of neoantigens being identified, it is time to think about the question of which characteristics are shared among a wide variety of cancer types, including the cancers with few mutations, so that cancer patients can receive the rational proposed therapeutics. Overall, the identification of neoantigens is a definite frontier in cancer research and will strengthen our understanding of essential targets in cancers and increase the stakes when we fight them.

## Author Contributions

ZW has completed all literature search, conceptual framework, diagrams and figures design, manuscript writing, editing, and proofing with the help of YC, who has taken the charge of manuscript revision.

### Conflict of Interest

The authors declare that the research was conducted in the absence of any commercial or financial relationships that could be construed as a potential conflict of interest.
